# Experiences of women who inject Nyaope residing in the City of Tshwane Municipality, Gauteng

**DOI:** 10.4102/hsag.v30i0.2760

**Published:** 2025-05-22

**Authors:** Moganki H. Lefoka, Robert T. Netangaheni

**Affiliations:** 1 Department of Family Medicine, Faculty of Health Sciences, University of Pretoria, Tshwane, South Africa; 2 Department of Health Studies, Faculty of Human Sciences, University of South Africa, Tshwane, South Africa

**Keywords:** Nyaope, harm reduction, women who inject drugs, women who use drugs, needle and syringe programme

## Abstract

**Background:**

Substance use disorder (SUD) is a serious public health concern in South Africa and throughout the world. Substance use disorder manifests itself as repeated use of a substance causes health problems and social dysfunction, such as health issues, impairment and failure to meet obligations. People who inject drugs (PWIDs) are a particularly vulnerable population with high rates of illness and early deaths. The experiences of women who inject Nyaope (WWIN) are not well understood because they are not often the subject of studies. Therefore, it is important to understand the experience of WWIN in order to understand their needs.

**Aim:**

This study explored the experiences of WWIN who reside in the City of Tshwane Municipality, Gauteng.

**Setting:**

The study was conducted in the Community-Oriented Substance Use Programme.

**Methods:**

A qualitative research method using exploratory and descriptive designs was employed. Twenty-four women, ages from 19 years – 35 years, with a history of injecting Nyaope were interviewed using a semi-structured interview guide. Data were analysed thematically.

**Results:**

Women who inject Nyaope engage in high-risk behaviours such as sex work, transactional sex, inconsistent condom use and sharing of needles. They further experience stigma in different social settings, like family, community, health settings and with the police, and they also self-stigmatise. They experience a variety of unmet needs.

**Conclusion:**

Women who inject Nyaope engage in high-risk behaviour and experience stigma across social settings, including self-stigma, and because of their lifestyle, they have a variety of unmet needs.

**Contribution:**

There is a scarcity of literature on WWIN in a South African context. The findings add to the existing literature on WWIN.

## Introduction

Substance use disorder (SUD) is a serious public health concern in South Africa (SA) and throughout the world (Nzaumvila, Mash & Helliwell 2023). Based on statistical data, South Africa’s drug use is highest in Africa and almost twice as high as it is worldwide (Charlton, Negota & Mistry 2019; Ratshisusu et al. 2024). Substance use disorder has presented itself as an overwhelming challenge; it is a concern that threatens humanity’s social cohesion and negatively affects the lives of both users and non-users (Charlton et al. 2019). Substance use disorder is a term that encompasses both substance abuse and substance dependence (SAMHSA 2011), and it manifests itself when repeated use of a substance causes health problems and social dysfunction, such as health issues, impairment and failure to fulfil their roles (Rawat, Petzer & Gurayah 2021).

It is worth noting that, in SA, the number of people affected by SUD, including that of women, is increasing in communities, despite the efforts by the Department of Social Development (DSD), various stakeholders and communities (Mahlangu & Stephan 2018). Recent literature (Bala & Kang’ethe 2021; Scheibe, Shelly & Stowe 2024) has confirmed a steady increase in drug consumption in SA, with Nyaope leading the pack.

Nyaope is a highly potent drug compared to other well-known drugs (Varshney et al. 2023), and it is commonly used in South Africa (Ratshisusu et al. 2024), and its full composition is generally not known (Nzaumvila et al. 2023), although most agree that heroin is the main ingredient, and rumours of the inclusion of Antiretroviral (ARVs), milk powder, rat poison, bicarbonate of soda and pool cleaner (Nzaumvila et al. 2023) have been documented. There is a dearth of analytical chemistry and forensic science literature on the drug ‘Nyaope’ (Mthembi, Mwenesongole & Cole 2018).

Nyaope is highly addictive, and once one becomes dependent on the drug, it is difficult to quit (Ratshisusu et al. 2024). It has devastating effects at the individual, family and community levels (Nzaumvila et al. 2023). It is reported that when users attempt to stop using Nyaope on their own, they experience severe withdrawal symptoms such as the appearance of flu-like symptoms, nausea, severe cramps, cold chills, frequent sweating and constant diarrhoea (Varshney et al. 2023). Nyaope is one of the cheapest illicit drugs available in South Africa (available for ±$3.00) and is easily accessible to many young people (Fernandes & Mokwena 2020). It is projected that about 15% of South African youth, including young women, are prone to engage in drug use, and the prospects of coming into contact with Nyaope are higher (Lefoka & Netangaheni 2022; Ratshisusu et al. 2024).

There are an estimated 3.2 million women who inject drugs (WWID) around the world, which makes up 20% of all drug users (Harm Reduction International 2020). As compared with South Africa, there are an estimated 76 000 people who inject drugs (PWIDs), and between 16% and 23% of these people are women (HRI 2020). These figures are likely to be underestimated when the concealing effects of criminalisation, gender power imbalances and stigma are considered (Shirley-Beavan et al. 2020).

Women who use drugs in South Africa face several challenges in their daily lives (Rigoni 2021). They are more likely than men to experience severe negative effects of substance use, which include increased drug dependence, financial difficulties, health concerns and engagement in high-risk behaviours connected to the human immunodeficiency virus (HIV) (Shirley-Beavan et al. 2020). Women are more vulnerable because of a multitude of environmental, societal and personal factors that can impact their capacity to participate in health-promoting activities like harm reduction (Shirley-Beavan et al. 2020).

Women clearly need substance use services, yet the literature (Schamp, Vanderplasschen & Meulewaeter 2022; Shirley-Beavan et al. 2020) reveals that those who are in charge of developing SUD services develop the services without considering the needs of women who use drugs. Therefore, masculinist concerns still dominate the available services, failing to adequately meet the needs of women. There is a wealth of evidence showing that women who take drugs are not often the subject of research, which leaves their experiences poorly understood (El-Bassel & Strathdee 2015; UNAIDS 2014).

Furthermore, women who use drugs are stigmatised for their drug use behaviour. Society has certain perceptions of what is normal and what is not. People who are considered different from normal are stigmatised, perceived as deviant and often marginalised (Lee & Boeri 2017). Stigma is a process of labelling, stereotyping, social rejection, prejudice, rejection, ignorance, status loss, low self-esteem, low self-efficacy, marginalisation and discrimination, as well as the internalisation of community attitudes in the form of shame by person and family (Subu et al. 2021).

To develop an in-depth understanding of the experiences of women who inject Nyaope (WWIN), this study sought to explore and describe the experiences of WWIN residing in the City of Tshwane (CoT) Municipality. Consequently, the findings may assist policymakers in the field of substance use, researchers and programme implementers to clearly understand the experience of WWIN.

### Research question

The study was guided by the following research question: *What are the experiences of women who inject Nyaope residing in the City of Tshwane Municipality, Gauteng?*

### Study purpose

The study aimed to explore the experiences of WWIN residing in the CoT Municipality, Gauteng.

## Research methods and design

### Study design

A qualitative research method using exploratory and descriptive designs was employed. The emphasis of the qualitative research method was on exploring and describing the experience of WWIN residing in the CoT Municipality. Qualitative research is used to answer questions about people’s experiences, perspectives and meanings based on their own understanding. Qualitative research was deemed appropriate because it allows the researcher to delve into the lives of WWIN.

### Research setting

The study was conducted in the Community-Oriented Substance Use Programme (COSUP) located within the CoT Municipality. Community-Oriented Substance Use Programme was selected as an ideal research setting, as it provides comprehensive harm reduction services to people who use drugs, including WWIN.

### Study population and sampling strategy

Women living in the CoT Municipality who were above 18 years old and who had injected Nyaope for at least 6 months or more were considered as the study’s population. The participants were recruited from clients receiving substance use-related services at COSUP. The purposive sampling approach was used to sample participants. Interviews were conducted with nine women who had previously injected Nyaope and 15 women who were currently injecting Nyaope. Inclusion and exclusion criteria were adhered to during the sample procedure. Six months of Nyaope injection was thought to be enough time to assess a person’s level of experience, and individuals with fewer months were excluded from participating in the study.

### Inclusion criteria

The participant must identify as female with a history of injecting Nyaope for 6 months or more; they must be a resident of the CoT Municipality and be 18 years of age or older.

### Exclusion criteria

The participants who were not the residents of CoT Municipality, younger than 18 years, not identifying as females and intoxicated during the data collection were excluded.

### Data collection

Community-Oriented Substance Use Programme management granted the researchers permission to access research participants within the COSUP organisation and to refer participants who might need a debriefing session to their internal social workers if the participants need so after the interview. For the purpose of gathering data, the researcher liaised with the site social workers. All participants were provided with informed consent to participate in the study. The participation was voluntary, and participants were not coerced to participate in the study.

Semi-structured interviews were used by the researcher to collect data from 24 participants. Every interview was audio-recorded and transcribed. Confidentiality and information protection measures were taken to ensure the safety of the recordings and transcripts. In contrast to the researcher’s initial plan to interview 30 participants – 15 recovered injectors and 15 current injectors – only 15 current injectors and nine recovered injectors were interviewed. The researcher stopped the interviews after reaching data saturation. Data saturation is the phase in data collection where a researcher is collecting very little or no new information from the research participants (Kumar 2014). Data saturation was attained after the 19th interview, but the researcher continued until the 24th interview. Five additional interviews were done after data saturation because the researcher wanted to ensure that the interview process was not terminated prematurely. The data collection period started on the 01 July 2019 and came to completion on the 26 July 2019.

### Data analysis

Data were analysed using six phases of thematic analysis as identified by Braun and Clarke (2006). The first phase was to become acquainted with the content of the transcripts. The second phase involved examining each transcript, discerning preliminary codes that enabled them to categorise numerous codes that could potentially constitute distinct themes. The third phase was concerned with identifying potential themes; the researchers first grouped together codes that had similarities. Lastly, the researchers carefully arranged the codes and themes in a tabular manner. The fourth phase involved reviewing the themes and accommodating complementing themes. The fifth phase involved establishing what each theme covered and what kinds of data it included, and lastly, the sixth phase involved producing a theme report.

### Measures of trustworthiness

The standard of good qualitative research is based on trustworthiness. The researcher ensured trustworthiness by employing four constructs of trustworthiness, namely, credibility, transferability, dependability and conformability (Kumar 2014). Credibility in this study was ensured through: (1) building rapport with participants. Pre-counselling was conducted with all participants before data collection. (2) Transcript verification: transcripts were taken back so that the participants could establish if the interview transcript did not lose meaning during the translation process. (3) Two participants who were under the influence of the substance(s) at the time of the interview were not considered.

Transferability was established. The researcher has interviewed multiple participants from different areas within the CoT Municipality to ensure thick descriptions of data. Furthermore, the researcher has audiotaped the interview and observed and explored nonverbal messages during the interview. Conformability was captured by audio recording of the participants, following up on questions, probing and not assuming what the researcher did not understand. To ensure dependability, triangulation was utilised by conducting face-to-face interviews, using a digital voice recorder, taking field notes and allowing the research supervisor to assist with co-coding.

### Biographic characteristics of the participants

Table 1 contains the biographical characteristics of the participants.

**TABLE 1:** Research participants.

Participant number	Employment status	Age (years)	Ethnicity	Duration of injecting Nyaope (years)	Duration without using drugs
Participant 1	Unemployed	23	Black people	7	Current injector
Participant 2	Unemployed	32	Coloured people	4	Current injector
Participant 3	Unemployed	34	Black people	5	Current injector
Participant 4	Unemployed	22	Black people	4	Current injector
Participant 5	Unemployed	31	Black people	5	Current injector
Participant 6	Unemployed	27	Black people	6	Current injector
Participant 7	Unemployed	24	Black people	8	Current injector
Participant 8	Unemployed	35	Black people	13	Current injector
Participant 9	Unemployed	31	Black people	11	Current injector
Participant 10	Unemployed	32	Black people	12	Current injector
Participant 11	Unemployed	35	Coloured people	9	Current injector
Participant 12	Unemployed	32	Coloured people	4	Current injector
Participant 13	Unemployed	28	Coloured people	1	Current injector
Participant 14	Unemployed	23	Black people	4	Current injector
Participant 15	Unemployed	27	Black people	9	Current injector
Participant 16	Unemployed	31	Black people	6	5 months
Participant 17	Unemployed	27	Black people	10	1 year 8 months
Participant 18	Unemployed	32	Black people	6	8 months
Participant 19	Unemployed	30	Black people	9	4 months
Participant 20	Unemployed	35	Black people	16	5 months
Participant 21	Unemployed	36	Black people	6	6 years
Participant 22	Unemployed	32	Black people	15	9 months
Participant 23	Unemployed	31	Black people	12	5 months
Participant 24	Unemployed	29	Black people	9	11 months

*Source:* Lefoka, M.H., 2019, ‘Exploring the experiences of women injecting Nyaope residing in the City of Tshwane Municipality, Gauteng’, Dissertation for Masters of Social Behavioural Studies in HIV/AIDS, University of South Africa

### Ethical considerations

Ethical approval to conduct this study was obtained from the University of South Africa College of Human Science Research Ethics Review Committee (Ref no: 2019-CHS-0246). All participants provided informed consent to participate in the study. The participation was voluntary, and the researcher adhered to social science ethics throughout the study.

## Results

Data were collected from a sample of 24 women with a history of injecting Nyaope. Three main themes, 13 sub-themes and three categories emerged from the data analysis (Table 2).

**TABLE 2:** Themes, sub-themes and categories.

Themes	Sub-themes	Categories
1. Risky behaviour	1.1 Sex work	-
	1.2 Transactional sex	-
	1.3 Inconsistent condom use	-
	1.4 Sharing needles	-
2. Social interaction	2.1 Interaction with the family	-
	2.2 Interaction with the community	-
	2.3 Interaction with the health care institutions	2.3.1 Attitude of health care workers 2.3.2 Sexual reproductive health knowledge gap 2.3.3 Access to prenatal care
	2.4 Interaction with the police	-
	2.5 Interaction with self (Self-perception)	-
	3. Needs of women who inject Nyaope	3.1 Basic needs	-
		3.2 Dreams and aspirations	-
3.3 Affordable drugs		-
3.4 Access to needles		-

### Theme 1: Risky behaviour

Participants reported to have participated in risky behaviours which have the probability of exposing them to HIV and other blood-borne infections. Sex work, transactional sex, inconsistent condom use and needle sharing were reported by the participants.

#### Sub-theme 1.1: Sex work

Five participants stated that they make money from sex work. Sex work is regarded as a viable source of income as opposed to relying on others to fund their drug use. This statement is supported by the following quotes:

‘I sell my body.’ (Participant 2, Female, 32 years old)

‘I am hustling [*making money*] through sex work.’ (Participant 6, Female, 27 years old)

‘At times my boyfriends would give me [*Money*], but he began to complain, so I looked for other options then I became a sex worker, so that I can be able to buy myself drugs.’ (Participant 24, Female, 29 years old)

#### Sub-theme 1.2: Transactional sex

Twelve participants reported engaging in transactional sex to fund their drug use. They are engaging in this practice without the knowledge of their intimate partners. The participants reported that:

‘Truly speaking, even my boyfriend does not know. I have people who I meet secretively, and I would give them sex in exchange of money.’ (Participant 15, Female, 27 years old)

‘Sometimes I sleep with someone for money.’ (Participant 10, Female, 32 years old)

‘Since I started using Nyaope I do not have feelings, I do not like to be intimate with men, but I do it, even if a man can come with R300 and say sleep with me I will do it because I want to buy drugs. I need that money.’ (Participant 8, Female, 35 years old)

Engaging in substance use requires money, and one is required to continuously raise money to be able to sustain their drug use. Participants have reported engaging in transactional sex to raise funds. Transactional sex involves engaging in sexual activity with different partners, which increases the risk of contracting HIV and other blood-borne infections.

#### Sub-theme 1.3: Inconsistent condom use

When it comes to discussing condom use with their intimate partners, participants expressed feeling powerless and disempowered, suggesting that they have given up control over their health to their partners. Some participants shared fear of provocation, which might have led to physical violence as a factor to allow their partner to have their way. Fourteen participants reported having engaged in inconsistent condom use with their sexual partners. This is supported by the following statements:

‘You know when you talk to males about using condoms they do not understand, at times they want to have unprotected sex. I think that we were going to fight maybe he was going to hit me.’ (Participant 24, Female, 29 years old)

‘It is hard to refuse my partner as he would accuse me that I have been sleeping around, why I am refusing? It is difficult because I do refuse at times and suggest condom use, but he does not want to.’ (Participant 20, Female, 35 years old)

#### Sub-theme 1.4: Sharing needles

Needle sharing is a common practice within the social network of PWIDs because of the unbearable withdrawal symptoms associated with Nyaope. Participants reported that not having access to clean needles has made them share needles because they don’t consider the risks involved when withdrawing from Nyaope. When they withdraw, the focus is on clearing drug withdrawals, and they tend to overlook the risk associated with sharing needles. Nineteen participants reported to have shared needles within their injecting network. The participants reported that:

‘When you experience withdrawal you lose your mind, you forget about everything, you forget about sickness, you forget that what you are doing will get you in trouble and you will remember after.’ (Participant 5, Female, 31 years old)

‘I was not thinking about all that [*the risk of sharing needles*], my main concern was to smoke [*to inject*] and relieve myself from the withdrawal symptoms and be high.’ (Participant 24, Female, 29 years old)

Additionally, participants ignored the risks of sharing needles when sharing with their intimate partner. Participants reported:

‘I just said because is my boyfriend, I only shared with my boyfriend.’ (Participant 1, Female, 23 years old)

‘He did not have an injection, actually we had but it blocked, so he asked if he can use mine.’ (Participant 10, Female, 32 years old)

### Theme 2: Social interaction

The participants are affected by various factors through interacting with their families, community, health care institutions, police and themselves.

#### Sub-theme 2.1: Interaction with the family

All 24 participants reported that when their relatives discovered that they were smoking Nyaope, they were unhappy with them. Families experienced a range of negative feelings, including astonishment, anger and disappointment, upon discovering that participants were using Nyaope. This is supported by the following statements:

‘They were really disappointed, shocked, because I was the brilliant child in the family.’ (Participant 18, Female, 32 years old)

‘They were so angry, and their hearts were broken, it contributed to conflicts in the family, it was not nice.’ (Participant 9, Female, 31 years old)

Three participants reported that even though their families were first angry, then their families extended their hand to help them when they learnt that they use drugs. The supportive nature of the family was revealed by the efforts to help the participants at different institutions. This is supported by the following narratives:

‘They tried everything, first they took me to places where they thought they will help me to stop, even to church and then I came to COSUP.’ (Participant 14, Female, 23 years old)

‘She is a parent; no parent wants their child to do wrong things. She tried to take me to rehab and took me to another doctor, they inserted another pill [*Naltrexone*].’ (Participant 19, Female, 30 years old)

‘My mother tried to help me, even before she passed away, she went some place for me to get help but before she could, she passed away.’ (Participant 22, Female, 32 years old)

Fourteen participants reported that after their families discovered that they had transitioned from smoking to injecting Nyaope, the reactions of the families worsened from how they reacted when they discovered that the participants were smoking Nyaope. The families associate Nyaope injecting with suicide. Participants reported:

‘My mother was worried thinking that I am killing myself, saying that I will die very young.’ (Participant 24, Female, 29 years old)

‘They were not happy, they were not happy at all, they thought I’m going to die.’ (Participant 2, Female, 32 years old)

#### Sub-theme 2.2: Interaction with the community

The community discriminates and stigmatises women for using Nyaope. Fourteen participants reported that the way they related with their community before using Nyaope changed drastically. The community changed how they treated them. Participants reported that they became untrustworthy and were stigmatised because of drug use. Participants reported:

‘They started acting up, others would distance themselves from me, and others would not greet me. They do not trust me, even if a person wants to ask me to run their errands, it becomes difficult because of they might think that I will run away with their money.’ (Participant 23, Female, 31 years old)

‘The community, to be honest, once they know you smoke or injection nyaope they start to look down upon you. They do not take you as a person anymore, to them you are just a nothing.’ (Participant 1, Female, 23 years old)

Eight participants reported that some community members showed love and support in place of the typical discrimination that is directed towards women who use Nyaope. The participants reported that:

‘Some treat me well and others discriminate against me and undermine me. Others changed, sometimes they treat you well, sometimes they stigmatise.’ (Participant 9, Female, 31 years old)

‘They did not discriminate against me … they were supporting me, they would give me advice, they even offered to buy me drugs … they only emphasised that they do not want me to steal because of nyaope.’ (Participant 3, Female, 34 years old)

Transition from smoking Nyaope to injecting attracted intense stigmatisation from the community. The community, like the family, equates transition to suicide. They perceived it as an extremely risky behaviour. Eleven participants reported to have experienced stigmatisation after the community discovered that they have transitioned from smoking to injecting. The participants have reported that:

‘Yoh! They treated me badly, very bad. When I pass by, they would say it is that girl who injects nyaope, what kind of a girl is that; people would talk behind, and I hear them.’ (Participant 24, Female, 29 years old)

‘They are saying now they are killing themselves, this is the end, and some are happy, someone said few weeks back.’ (Participant 9, Female, 31 years old)

#### Sub-theme 2.3: Interaction with the health care institutions

Participants shared their experiences when interacting with health care institutions. They explored the attitudes of health care workers, their knowledge on their sexual reproductive health as women who use drugs and those who got pregnant while using drugs; they reported on the history of their access to prenatal care.

***Category 2.3.1: Attitude of health care workers*:** Fifteen participants highlighted that they experience stigma when accessing health care services. They reported that once the health care professionals discover that the participants use drugs, their attitude towards participants changes. They reported that:

‘They treated you well, but when you tell them that you use Nyaope they change.’ (Participant 2, Female, 32 years old)

‘You do not feel welcomed as long as it is about withdrawals or if the nurses can find out that you are an addict, they do not treat you nice.’ (Participant 8, Female, 35 years old)

***Category 2.3.2: Sexual reproductive health knowledge gap*:** Participants reported a gap in sexual reproductive knowledge, which can be addressed by educating women who use Nyaope about their sexual reproductive health. The participants believe that because they do not observe their menstrual cycles due to Nyaope use, the likelihood of them getting pregnant is slim or nonexistent. This misconception can be addressed if participants were accessing sexual reproductive health services at their local clinics. Sixteen participants did not attempt to access sexual reproductive health services, while eight participants were accessing sexual reproductive health services. Participants who were not accessing sexual reproductive health services reported that:

‘When I use drugs, I do not fall pregnant. I am telling you, but when I do not use drugs and my system is clean, I fall pregnant easily.’ (Participant 12, Female, 32years old)

‘When smoking, the chances of getting pregnant are slim, because I do not go to periods [*menstruation cycle*].’ (Participant 17, Female, 27 years old)

‘I did not have a regular menstrual period. Even now I have not gone on the periods. What I know is that when smoking nyaope as a woman, it becomes difficult to go on the menstrual cycle.’ (Participant 23, Female, 31 years old)

***Category 2.3.3: Access to prenatal care:*** Two participants got pregnant while using drugs, and they only knew about their pregnancy until quite late, making it more challenging for them to have an opportunity to benefit from prenatal care, harm reduction services, drug treatment and other support, or to terminate their pregnancies safely if they so choose. Participants reported that:

‘I did not even know that I was pregnant, I found out when I was 5 months, and on the 7th month I delivered.’ (Participant 8, Female, 35 years old)

‘I was 7 months pregnant when I discovered my pregnancy.’ (Participant 19, Female, 30 years old)

One participant who became pregnant did not access prenatal care services when she was pregnant. The participant reported that:

‘I did not attend the clinic until the child came. The child came before time, but he is fine, healthy, and well. I was scared that the child can be disabled or come deformed, but I was unable to stop.’ (Participant 8, Female, 32 years old)

#### Sub-theme 2.4: Interaction with the police

Eight participants have reported unpleasant experiences with the police. They have indicated that police have arrested them without being charged when they were found in possession of needles. Participants reported that this practice of being arrested by the police only to be released the following day without appearing before a magistrate is called ‘white door’ arrest. This is supported by the following statements:

‘Sometimes the police would they take us to holding cells and we remain in the police cell for 48 hours. After 48 hours, we are released through a white door.’ (Participant 22, Female, 32 years old)

‘I was arrested but I was out with white door, it is like you do not get to go to court; they keep you in police station.’ (Participant 7, Female, 24 years old)

Twelve participants further reported that the police will, in addition to arresting them, also break and confiscate their needles when they are found in possession of needles. Participants reported that:

‘They [*Police*] did not arrest me, they took my needles and broke them.’ (Participant 11, Female, 24 years old)

‘They [*Police*] took all of them [*the needles*], like, I just came from COSUP [*Needle and Syringe Programme {NSP} service provider*] to exchange needles, and they took all of them.’ (Participant 4, Female, 22 years old)

#### Sub-theme 2.5: Interaction with self (self-perception)

Eight participants looked down on themselves because they are women who use drugs. They believe that they don’t deserve to be loved. They are self-stigmatising, creating identities that make it impossible for them to have meaningful relationships:

‘I feel like a failure, but I do not want to put that mind of a failure to me because I want to fight this thing.’ (Participant 8, Female, 35 years old)

‘I just need to get out of this thing of drugs, it is not nice for a woman to use drugs.’ (Participant 11, Female, 35 years old)

‘[*I*]t makes me feel like I am a bad child I am giving my mother too much grieve at home.’ (Participant 12, Female, 32 years old)

### Theme 3: Needs of women who inject Nyaope

The participants reported a strong desire to meet a variety of needs in order to be functional.

#### Sub-theme 3.1: Basic needs

Eight participants highlighted the need to access the fundamental necessities for survival. They reported the difficulties they experience when they have to choose between purchasing drugs and essentials like toiletries. Drugs are expensive, and the challenge of choosing between basic needs and drugs is a dilemma. Participants acknowledge that because of using drugs, sometimes their decisions are not how many will approve. The participant reported:

‘I was not able to do what I want at the time I wanted, if I want to get my hair fixed or I want toiletry or pads, it becomes difficult to get those things … my priority would be to buy drugs.’ (Participant 23, Female, 31 years old)

‘Needed food, needed to bath, needed clothes, and I needed to live normal.’ (Participant 17, Female, 32 years old)

‘Maybe sometimes they can give us food, to get healthy foods, medication maybe if we are sick.’ (Participant 2, Female, 32 years old)

#### Sub-theme 3.2: Dreams and aspirations

Eighteen participants reported that they have dreams and aspirations to contribute positively in the lives of those they are close to. Drug use might have delayed and wasted their time, but they are determined to stop using drugs so that they can focus on improving their lives and that of their families. The participants indicated the need to be financially stable and independent:

‘To go back to school and upgrade my studies, get the job and also take care of my kids and family.’ (Participant 9, Female, 31 years old)

‘To get a job, look after my child, for God to bless me and get married get my own home and my own car.’ (Participant 18, Female, 32 years old)

A participant said that employers ought to be aware that she injects Nyaope and shouldn’t deny her employment because of her drug use. This view will be embraced by other women who use drugs and don’t have the intention of stopping to use drugs. It is supported by the following narrative:

‘I need a job, which can understand that I am using Nyaope and that I am injecting, because I need to come off the streets that will help me.’ (Participant 2, Female, 32 years old)

One participant, while sobbing, reported that it is better to work than to engage in sex work, where she will be required to sleep with different men to get 100. This is supported by:

‘So, it is painful to think that if now you want R100 you need to sleep with five people. But if one has a part time job that would give her R100 in a day it would be much better.’ (Participant 5, Female, 31 years old)

#### Sub-theme 3.3: Affordable drugs

Nyaope is not inexpensive, particularly when tolerance rises and spending goes up. One participant mentioned the necessity for reasonably affordable drugs. Drug affordability can lessen the everyday financial stress experienced by WWIN. She reported that:

‘[*T*]o have access to drugs or maybe to have them at the cheaper price. We can buy them for R10. I would really want to have access to drugs.’ (Participant 9, Female, 31 years old)

#### Sub-theme 3.4: Access to needles

Twenty participants highlighted a desire to access sterile needles. Accessibility of sterile needles is key as it can contribute towards sharing, should those who need them struggle to access them:

‘My needs are to get needles, at times I do not get them here, and you find that they are out of stock.’ (Participant 8, Female, 35 years old)

‘Access to clean needles.’ (Participant 1, Female, 23 years old)

## Discussion

The study’s overall findings add to the existing literature on WWIN and may be applicable to other drugs. The findings of the study provide an understanding of the risky behaviour WWIN engage in. Furthermore, the study sheds light on the experiences of WWIN while in interaction with the family, community, health care workers and police, and the unmet needs experienced by these women.

Participants reported engaging in sex work and transactional sex. Sex work and transactional sex are different concepts; sex work is understood to be a formalised and commercial negotiation of sex in return for payment, whereas transactional sex is described as informal, non-commercial and where love and trust may sometimes be present (Crankshaw & Freedman 2023). Additionally, women who use drugs and women who engage in sex work are two overlapping communities. Women who use drugs engage in sex work to support basic needs and drug use (Stanyard 2023). Larney et al. (2015) argue that women who use drugs are underprivileged or have limited job opportunities and are more likely to engage in sex work, which raises the risk of HIV and adds to the stigma associated with sex work.

Inconsistent condom use with intimate partners was reported. Participants reported a sense of helplessness and disempowerment in negotiating condom use with their sexual partners; this is linked to gendered cultural belief influence. Stoicescu et al. (2018) allude to the fact that social norms contribute to women prioritising maintaining harmony in relationships, putting one’s intimate partner above oneself and avoiding confrontation, which may make it harder for women to talk about safer sex, even when they feel unsafe.

The participants further reported that they have shared needles within their drug-injecting network. Needle-sharing practice is one of the main drivers of HIV transmission amongst PWID (The United Nations Office on Drugs and Crime 2019). Human immunodeficiency virus contact through used injecting equipment is six times more probable to transmit infection than contact through unprotected vaginal intercourse (World Health Organization 2015). Therefore, it is imperative that WWIN never run out of needles because, although they may be aware of the hazards of infection, their need for the drug during physical withdrawals outweighs all other considerations (Chakrapani et al. 2011). Access to sterile needles may reduce needle sharing among WWIN and their network, which may further reduce HIV incidence and curb other blood-borne infections. Participants further added that they shared needles with their intimate partners, ignoring the risks in the context of injecting intimate relationships.

The participants are affected and influenced by various factors that happen within their social context, and it is important to understand their social interactions. Social interaction can be viewed as encounters between at least two people in which they attend to one another and adjust their behaviour in response to one another (Hoppler, Segerer & Nikitin 2022). These studies looked at the family, community, health institutions, police and self. Stigma is the theme that is reflected across all social settings. Stigma is a process of labelling, stereotyping, social rejection, prejudice, rejection, ignorance, status loss, low self-esteem, low self-efficacy, marginalisation and discrimination, as well as the internalisation of community attitudes in the form of shame by person and family (Subu et al. 2021).

Families experienced lots of unpleasant emotions when they discovered that the participants were using Nyaope. They were shocked, angry, surprised and disappointed. This is in line with the finding of the study conducted by Schultz and Alpaslan (2016), which attests to the fact that the family’s first response upon discovering that one of its members is a drug user is astonishment; feelings of dismay, fury, frustration, sadness, confusion, helplessness, sympathy and disbelief accompany the shock. Furthermore, some participants reported that their relatives responded by providing them with support and escorting them to facilities where they might receive SUD assistance or receive rehabilitation. The family was dissatisfied with WWIN’s behaviour after finding out that they had transitioned from smoking to injecting. The family associates drug injection with suicide; the way they treated participants after discovering that they are now injecting Nyaope got worse than when they discovered they smoke Nyaope.

Research shows that WWID face more stigma than their male counterparts because injecting drugs is seen as a violation of women’s traditional duties in society as mothers, the anchors of their families and caretakers (El-Bassel & Strathdee 2015; Lee & Boeri 2017). Therefore, drug use brings shame and dishonour to one’s family, friends and community. Women who inject drugs indicated that they experienced stigmatisation at the hands of the community. The community changed how they treated them immediately when they learnt that they were using drugs. Some participants said that some community members showed love and encouragement in place of the typical discrimination against WWIDs.

Following the community’s discovery that the participants had transitioned from smoking to injecting, stigma and discrimination intensified. Research on stigmatisation in social and healthcare contexts conducted in India, Mexico, Vietnam and the United Kingdom has shown that social condemnation of women who use drugs often results in severe stigma and social exclusion from these societies for WWID (Mburu et al. 2018). Most drug users are now victims of police harassment and mob punishment because of the stigma attached to this substance (Bala & Kang’ethe 2021).

The participants highlighted unpleasant experiences when visiting health care centres because of stigma and discrimination. This is not unique to the South African context; Shirley-Beavan et al. (2020) contend that women who use drugs experience prevalent stigma across the health system. When health professionals discover about PWID’s drug use, stigma and prejudice have an impact on how they interact with and communicate with them (Carusone et al. 2019). It has been determined that stigma is a crucial impediment to PWID care, which results in poor health outcomes (Lan et al. 2018).

The participants have shown a knowledge gap about how drug use affects their menstrual cycle, which disadvantages them. It is important that programmes offering SUD services offer sexual and reproductive health services. These initiatives will enable women to take charge of their sexual and reproductive health, thereby improving pregnancy outcomes and reducing unwanted pregnancies (Pinkham & Malinowska-Sempruch 2008).

The participants have reported hostile experiences with the police; this finding is in line with other studies. El-Bassel and Strathdee (2015), in their study, also found out that WWUD are ill-treated by police officers. The participants said that they were arrested by police without being charged and that they had their needles broken and confiscated by the police. The Drug Master Plan (2019–2024) endorses the NSP (DSD 2019); the first South African National Strategic Plan for HIV, TB and STIs 2023–2028 further endorses NSP as a strategy to curb HIV among PWIDs. Confiscating of needles reverses the prevention efforts of those who implement NSP. Although drug use is illegal in South Africa, purchasing or supplying injecting equipment is not prohibited by law (UNODC 2017).

Participants also mentioned that they were taken into police cells by the police without receiving any formal charges. The term ‘white door arrest’ has been used by eight participants to describe this kind of arrest. The arrest is not unique to the CoT; Shirley-Beavan et al. (2020) have reported that people who use drugs (PWUD) are used by law enforcement as cover for mistreatment, employing aggressive policing tactics that include arresting women for carrying equipment for injection or smoking, planting drugs, harassing, asking for bribes, sexually abusing and abusing violence.

Participants have negative perceptions about themselves, which fuels self-stigma. The participants feel like failures, and they also feel that drugs are not meant for women. These stigma processes can prevent people from obtaining health care, causing them to put off seeking care, suffer low social support and its repercussions and refrain from thinking about their condition or loss of self-worth (Owczarzak et al. 2023).

Women who inject Nyaope experience several unmet needs because of their lifestyle. Participants’ access to necessities for survival is something that they desire, and they reported the difficulties they encounter when they have to purchase necessities like hygiene items as opposed to buying drugs. The DSD, through its partners, should establish drop-in centres to close the gap experienced by WWIN. A drop-in centre presents an opportunity to offer services that go beyond SUD services and address the more extensive physical and social needs of drug users (UNODC 2017).

Regardless of how they see themselves, WWIN have dreams and aspirations. They wish to fulfil independence and financial stability, which will empower them to regain social functionality, which, in turn, will help them feel better about themselves because it will enable them to make positive contributions to both their own and their significant others’ lives. People with a history of drug use are more likely to be able to maintain their recovery if they live in a safe environment and are self-sufficient financially (Stokes, Schultz & Alpaslan 2018). One participant said that employers ought to be aware of their drug use and should not discriminate against them because of their substance use.

Not everyone wants to stop using drugs; therefore, service providers should not presume people who use drugs desire to stop using. Because Nyaope is expensive, especially when tolerance rises, one participant mentioned the need for reasonably affordable drugs. Drug affordability may lessen the daily financial struggles WWIN encounter. Women who inject Nyaope want to have access to clean needles; they don’t want to share needles, and having clean needles available to them will help.

### Limitations of the study

The study employed qualitative research methods, and the findings cannot be generalised. Only participants who were using specific COSUP sites for service access were included in this study. Research on PWID in South Africa was scarce; in 2017, UNODC conducted the only study on female Nyaope injectors in the country. For literature, the researcher relied on research conducted internationally. The sample consisted of WWID regardless of whether they live with relatives, are jobless, homeless or work for a living.

### Recommendations

Women who inject Nyaope engage in high-risk behaviour, which may increase the likelihood of contracting HIV and other blood-borne infections. Government, health managers, healthcare institutions and healthcare providers should all play their specific roles in order to upscale HIV prevention and harm reduction programmes targeting WWIN, including other drugs. Because of the high level of stigma experienced by WWIN in the social context, it is critical that community-based organisations that provide substance abuse services include stigmatisation sensitisation workshops in their programmes for families, community members, police, health care workers and other relevant groups of people. In addition, programmes should be created with the purpose of empowering WWIDs with skills to participate in economic activities. It is critical that SUD programmes provide sexual and reproductive health care to WWID.

## Conclusion

Women who use drugs are at risk for HIV and other blood-borne diseases because of a variety of individual, societal and institutional factors. The study has highlighted that WWIN engage in high-risk behaviours such as sex work, transactional sex, inconsistent condom use and needle sharing. They further encounter stigma in different social settings such as family, community, health care settings and police, and they also self-stigmatise.

Women who inject drugs should be financially empowered to address sexual risk behaviour impacted by being obliged to participate in sex work to fund their own or their partner’s drug use, which may expose them to HIV infection through both unsafe sex and unsafe injections. All the efforts to curb HIV will remain fruitless until WWIN are empowered to be independent.
